# Nurse Managers' Toxic Leadership Behaviors and Their Influence on Nurses' Quality of Life: A Cross-Sectional Study

**DOI:** 10.1155/jonm/6862124

**Published:** 2025-10-26

**Authors:** Zarina Sarsenbay, Aikorkem Murat, Tolganay Kamzayeva, Zhangul Beishenbay, Saule Bukhanova, Anargul Kuntuganova, Ejercito Mangawa Balay-Odao, Jonas Preposi Cruz

**Affiliations:** ^1^Department of Medicine, School of Medicine, Nazarbayev University, Astana, Kazakhstan; ^2^Department of Biomedical Sciences, School of Medicine, Nazarbayev University, Astana, Kazakhstan

**Keywords:** nurse managers, nurses, nursing management, quality of life, quantitative study, toxic leadership

## Abstract

**Background:**

Leadership and organizational climate significantly impact nurses' effectiveness, especially in the high-stress environments typical of healthcare institutions. However, toxic leadership characterized by micromanagement and bias can negatively affect employee morale and diminish service quality.

**Aim:**

This study aimed to examine how nurse managers' toxic leadership behaviors influence the quality of life of nurses in Kazakhstan.

**Methods:**

This research employed a quantitative, cross-sectional, and correlational design. The study was conducted at the University Medical Center in Astana, Kazakhstan. A convenience sample of 313 nurses participated in the survey, which included a paper questionnaire with three parts: a participant information sheet, the “Toxic Leadership Behaviors of Nurse Managers Scale” (ToxBH-NM), and the “World Health Organization Quality of Life” assessments. The demographic characteristics, toxic leadership behaviors of nurse managers, and quality of life were analyzed descriptively. Multiple regression analyses were used to examine the impact of nurse managers' toxic leadership behaviors on nurses' quality of life.

**Results:**

The findings indicated a generally positive nurse managers' leadership behaviors (*M* = 1.55, SD = 0.83). More than half of the nurses reported an overall quality of life that they described as good (55.3%) and expressed satisfaction with their health (56.2%). Weak to moderate inverse correlations (*r = *0.16 to 0.38, *p* < 0.001) were found between toxic leadership behaviors and the quality of life of nurses. The four regression models were found to be significant, explaining 15.7% (*R*^2^ = 0.192, adjusted *R*^2^ = 0.157), 8.9% (*R*^2^ = 0.127, adjusted *R*^2^ = 0.089), 11.8% (*R*^2^ = 0.155, adjusted *R*^2^ = 0.118), and 16.0% (*R*^2^ = 0.195, adjusted *R*^2^ = 0.160) of the total variance in “physical health” (*F*_13_,_299_ = 5.47, *p* < 0.001), “psychological health” (*F*_13_,_299_ = 3.34, *p* < 0.001), “social relationships” (*F*_13_,_299_ = 4.21, *p* < 0.001), and the “environment domain” (*F*_13_,_299_ = 5.56, *p* < 0.001).

**Conclusion:**

Experiences of humiliating behavior from nurse managers were linked to a poorer quality of life among nurses. Factors such as marital status, the specific hospital where the nurses worked, and the toxic leadership behaviors of nurse managers significantly affected the nurses' quality of life.

**Implication for Nursing Management:**

These findings can serve as a foundation for developing programs that specifically address the toxic leadership behaviors identified in the study, which adversely affect nurses' quality of life.

## 1. Introduction

The role of nurse managers is crucial for effectively leading nursing teams and motivating them to provide quality patient care [1]. Nurse managers oversee nursing operations in patient care delivery and act as team builders. Administratively, they serve as a bridge between management and staff, ensuring compliance with policies and procedures while also addressing the immediate needs of nursing teams [2]. The importance of nurse managers has become even more pronounced with advancements in the healthcare system and the emphasis on quality assurance in health services [3]. However, due to the ever-evolving nature of the healthcare system, nurse managers in Kazakhstan face numerous challenges, including inadequate training, insufficient leadership skills, and pressure to meet increasing demands despite limited resources [4].

Toxic leadership, characterized by authoritarianism, excessive control, unfair task distribution, and aggressive behavior, significantly lowers nurse morale, contributes to emotional burnout, and increases staff turnover [5]. Such an environment undermines commitment to essential professional values such as patient care and teamwork [6]. Leadership and organizational climate are crucial for nurses' effectiveness, especially in the high-stress settings typical of healthcare institutions. Nurse managers who create supportive atmospheres enhance both the quality of care and staff well-being. Conversely, micromanagement and bias damage employee morale and the overall quality of services [5]. A positive climate boosts job satisfaction and professional engagement among nurses, while a toxic environment exacerbates burnout and lowers healthcare quality [7].

The quality of life for nurses, encompassing physical and mental health, job satisfaction, and work–life balance, is directly influenced by the organizational climate and leadership style [8]. Factors such as stress, emotional burnout, and fatigue can negatively impact this quality of life [8]. However, support from leadership, recognition of achievements, and opportunities for professional growth can significantly enhance nurses' emotional and physical well-being, leading to better health and higher engagement in their work [9].

In Kazakhstan, the influence of leadership on subordinates is linked to traditional values that emphasize hierarchy, respect for elders, and family relationships [10]. While globalization and Western management models are effecting changes, Kazakhstani managers and employees often maintain their commitment to traditional approaches, such as paternalistic leadership, which encourages kind interactions [10]. This creates a conflict between modern leadership theories and local cultural norms, impacting subordinates' behavior and organizational culture. Despite Kazakhstan's inclination toward softer, more respectful leadership styles, toxic elements can still infiltrate the organizational environment, leading to subtle yet damaging consequences for employee well-being.

Currently, there are no studies specifically examining “toxic leadership in Kazakhstan,” as evidenced by the lack of relevant publications in international journals and specialized platforms. This highlights the relevance of investigating this phenomenon, particularly in the healthcare sector. The impact of toxic leadership styles on nurses' quality of life in Kazakhstan warrants special attention, as negative leadership behaviors can significantly deteriorate workers' psychological states and decrease productivity. Therefore, it is essential to explore the influence of toxic leadership on the quality of life for nurses in Kazakhstan.

### 1.1. Aim

This study aimed to examine the influence of nurse managers' toxic leadership behavior on the quality of life of nurses in Kazakhstan.

## 2. Methods

### 2.1. Design

This study is a quantitative study, specifically a cross-sectional and correlational study, to examine the influence of nurse managers' toxic leadership behavior on nurses' quality of life.

### 2.2. Samples and Settings

The study was conducted at the University Medical Center (UMC) located in Astana, Kazakhstan. This hospital operates three clinics: the Maternal and Child Center, the Heart Center, and the Diagnostic Center. UMC has a capacity of 778 beds and accommodates 500 out-patient clinic visits per shift. It provides health and medical services across various specialties, including obstetrics and gynecology, pediatrics, pediatric surgery, traumatology, urology, endocrinology, pulmonology, gastroenterology, neurology, cardiology, and cardiac surgery, among others. In addition, UMC is involved in educational and research activities and serves as a training hospital for students in health-related fields, such as medical and nursing students.

For sample selection, a convenience sampling technique was employed. The study's participants were identified based on the following inclusion criteria: (1) staff nurses currently working in the three clinics (Maternal and Child Center, Heart Center, and Diagnostic Center), (2) a minimum of 6 months of work experience at the hospital, (3) work in in-patient and out-patient departments, (4) provision of direct nursing care to patients, and (5) willingness to participate in the study. The exclusion criteria included the following: (1) nurses in administrative or managerial positions, (2) nurses who were on leave during the data collection period, and (3) participation in the pilot testing of the survey.

A priori power analysis was conducted to determine the minimum sample size required. Using G∗Power Version 3.1, the analysis revealed that the minimum sample size for a multiple linear regression, with a hypothesized effect size of 0.15, an alpha error probability of 0.05, 90% statistical power, and 13 predictor variables, was 162. Therefore, the current sample size of 313 is more than sufficient to ensure reliable results. To ensure an adequate sample size, the researchers approached 350 nurses who met the inclusion criteria during the data collection period. Out of these, 324 agreed to participate. However, 11 nurses returned the survey unanswered and were therefore excluded from the sample. This resulted in a response rate of 89.4%.

### 2.3. Instrument

Data collection was conducted using a paper-based survey that consisted of three parts: the participants' information sheet, the “Toxic Leadership Behaviors of Nurse Managers Scale” (ToxBH-NM) [5], and the “World Health Organization Quality of Life, BREF” (WHOQOL-BREF) [11].

The participants' information sheet gathered sociodemographic and work-related information from the participants. The variables included the following: (1) age, (2) gender, (3) marital status, (4) highest educational qualification, (5) the hospital where they work, (6) total years of experience as a nurse, and (7) years of experience in their current hospital. Total years of experience as a nurse were defined as all years worked as a nurse including prior to joining the current hospital.

The ToxBH-NM was used to assess the toxic behaviors of nurse managers. This is the only existing tool that specifically measures the toxic leadership behavior of nurse managers, which was the main variable of interest in this study. This scale contains 30 items with 5-point Likert scale response options (1 = “not at all,” 2 = “once in a while,” 3 = “sometimes,” 4 = “fairly often,” and 5 = “frequently”). It comprises four subscales: “intemperate behaviors” (15 items), “narcissistic behaviors” (9 items), “self-promoting behaviors” (3 items), and “humiliating behaviors” (3 items). “Intemperate behaviors” refer to hostile verbal or nonverbal actions by nurse managers toward their staff nurses. “Narcissistic behaviors” are actions driven by personal ambitions and self-absorption. “Self-promoting behaviors” involve actions taken by nurse managers to further their own personal or professional growth. Mean scores were computed for each subscale and for the entire scale. High mean scores indicate more frequent nurse managers' toxic behavior experienced by the nurses. The scale has demonstrated acceptable validity and reliability, with an overall Cronbach's alpha of 0.975, and a range of 0.895–0.965 for its four subscales. The test–retest reliability of the scale was determined to be 0.801, indicating excellent stability. Exploratory factor analysis supported the four-factor structure of the scale, explaining 71.84% of the variance [5].

To ensure accuracy and cultural relevance, the tool was translated into Russian and Kazakh, following the cross-cultural adaptation recommendations outlined by Beaton et al. [12]. The adaptation process included the following: (1) “Translation,” (2) “Synthesis,” (3) “Back Translation,” (4) “Expert Committee Review,” and (5) “Pretesting.” A group of 5 nursing experts, including two faculty members specializing in healthcare leadership and management, and three nurse managers, evaluated the content validity index (CVI) of the two translated versions. The item-level CVI values for both versions were 1, and the scale-level CVI, calculated using averaging techniques, was also 1. Both the Kazakh and Russian versions were piloted separately among 30 Kazakh nurses. The results indicated an overall Cronbach's alpha of 0.98 for the Kazakh version (“intemperate behaviors” = 0.96, “narcissistic behaviors” = 0.93, “self-promoting behaviors” = 0.91, and “humiliating behaviors” = 0.90) and 0.88 for the Russian version (“intemperate behaviors” = 0.80, “narcissistic behaviors” = 0.78, “self-promoting behaviors” = 0.93, and “humiliating behaviors” = 0.54). The participants in the pilot tests did not raise any concern about the tools; hence, no revisions were made. In the current sample (Kazakh version n = 100; Russian version n = 213), the calculated Cronbach's alpha for the Kazakh version was 0.96 (with subscale alpha values ranging from 0.80 to 0.93) and 0.98 for the Russian version (with subscale alpha values ranging from 0.92 to 0.97). The use of the tool was permitted by L. Labrague (personal communication, August 27, 2024).

The WHOQOL-BREF is a shorter version of the WHOQOL–100, designed to assess an individual's position in life within the context of their culture and value systems, as well as in relation to their goals, expectations, standards, and concerns. This instrument comprises 26 items with 5-point Likert scale response options. The WHOQOL-BREF evaluates the following domains: “physical health” (7 items), “psychological health” (6 items), “social relationships” (3 items), and “environment” (8 items). The first item measures the overall perceived quality of life, while the second item assesses overall perceived health. Each item is scored from 1 to 5 on the response scale. Raw domain scores for the WHOQOL were transformed to a 4–20 scale following established guidelines, and then linearly transformed to a 0–100 scale, where higher scores indicate better quality of life. The WHOQOL-BREF has shown excellent validity and reliability in various studies [11, 13]. The WHOQOL-BREF is a widely used tool in assessing the quality of life of various groups of individuals, including nurses (e.g., [14, 15]). The tool has also been used to measure the quality of life of nurses in Kazakhstan [16]. Hence, we have decided to use this tool in our study. The Kazakh and Russian versions of the tool were obtained from the WHO and utilized in this study. Both versions were piloted separately among 30 nurses, resulting in an overall computed Cronbach's alpha of 0.85 for the Kazakh version (subscale alpha range from 0.65 to 0.72) and 0.92 for the Russian version (subscale alpha range from 0.66 to 0.89). For the current sample (Kazakh version *n* = 100; Russian version *n* = 213), the Cronbach's alpha for the Kazakh version was 0.91 (Cronbach's alpha range of the subscales = 0.70–0.83) and 0.93 for the Russian version (Cronbach's alpha range of the subscales = 0.77–0.86). Permission to use the tool in this study was granted by the WHO (request ID: 202403368).

### 2.4. Recruitment and Data Collection

Participants were recruited face-to-face in hospitals. Researchers coordinated with the hospitals to determine the best schedule for data collection. They approached nurses during their break times or when they were not busy, inviting them to participate in the study. A recruitment script was prepared and read to the nurses, providing complete information about the study to ensure consistency in the recruitment message for all potential participants. After signing the informed consent, participants were given the survey and sufficient time to complete it. Researchers then collected the completed questionnaires and stored them in a locked cabinet for safekeeping. Data collection took place from February to March 2025.

### 2.5. Statistical Analysis

The analyses were conducted using SPSS Version 24.0. The demographic characteristics, toxic leadership behaviors of nurse managers, and quality of life were analyzed descriptively. Pearson's product–moment correlation was used to examine the relationships between the manager's toxic behaviors and the nurses' quality of life. This analysis was chosen as we wanted to test if relationships existed between managers' toxic behaviors and the nurses' quality of life. Moreover, the assumptions of conducting this test, such as normality and level of measurement, and independence of observations were met by our data. Multiple linear regression analyses were performed to assess the impact of the nurses' demographic characteristics and their manager's toxic leadership behaviors on their quality of life, allowing for the simultaneous evaluation of multiple predictors and the estimation of their independent contributions.

In the four regression models, the demographic variables and the four dimensions of the ToxBH-NM were included as predictor variables for each dimension of the WHOQOL-BREF. Regression coefficients (*β*), standard errors, and 95% confidence intervals were reported. Categorical or nominal predictor variables were dummy-coded before being entered into the regression models. The assumptions for conducting regression analysis (normality, linearity, multicollinearity, and homoscedasticity) were tested and met for each model. Statistical significance was defined as a p value of less than 0.05.

### 2.6. Ethical Considerations

The study protocol received approval from the Nazarbayev University School of Medicine Institutional Research Ethics Committee (NUSOM-IREC; Approval number: 2024NOV#02) and the Local Bioethics Committee of the UMC. Researchers adhered to the ethical guidelines set by both committees and followed the Declaration of Helsinki principles. To protect participants' health, dignity, and privacy, they were thoroughly informed about the study's purpose, procedures, risks, benefits, and their right to withdraw at any time without consequences. Participants had the opportunity to ask questions, and those who consented were required to sign an informed consent form to indicate their voluntary participation. No identifying information was collected, and data were reported in aggregate form. Completed surveys were stored securely, and data will be deleted as per the NUSOM-IREC policy.

## 3. Results

### 3.1. Demographic Characteristics

Table 1 provides an overview of the demographic characteristics of the participants. The ages of the nurses ranged from 19 to 64 years, with a mean age of 33.08 years (SD = 11.62). The majority of participants were female (96.2%), and most held a certificate in nursing or midwifery (60.1%). Nearly half of the participants were single (45.7%), while 39.6% were married and 14.7% were separated, divorced, or widowed. The largest group of participants worked at Hospital 3 (46.3%), followed by Hospital 2 (40.6%), and the smallest proportion was from Hospital 1 (13.1%). The mean of the total years of experience as a nurse was 11.06 years (SD = 11.06), and the mean years of experience at the current hospital was 6.01 years (SD = 6.08).

### 3.2. Nurse Managers' Toxic Leadership Behavior

The overall mean score of the participants on the ToxBH-NM scale was 1.55 (SD = 0.83), indicating a generally positive leadership behavior of their nurse managers. Among the four subscales of the ToxBH-NM, nurses reported the highest mean score for “Humiliating behavior” (*M* = 1.61, SD = 1.02), followed closely by “Narcissistic behavior” (*M* = 1.55, SD = 0.86) and “Intemperate behavior” (*M* = 1.54, SD = 0.83). The subscale “Self-promoting behavior” received the lowest mean score at 1.51 (SD = 0.93).

While the highest percentage of participants indicated “not at all” when asked if the behaviors described in the scale applied to their nurse managers (with the percentage reporting “not at all” ranging from 58.1% to 81.2%), some toxic behaviors were noted by a significant number of nurses at least occasionally. For instance, 41.9% of nurses observed their nurse managers raising their voices when their opinions were not favored or accepted by the staff. In addition, 36.7% reported that their nurse managers repeatedly reminded staff of their previous mistakes.

Around 33.5% of nurses experienced being belittled by their nurse managers at least sometimes, and 34.2% noted that their manager punished the entire unit for mistakes made by a single staff member at least occasionally. Furthermore, 33.2% of nurses felt that their manager believed they fully deserved their position, and 31.3% reported that their nurse manager thought they were always right at least sometimes. In addition, 31.6% of the participants observed that their nurse manager had a group of dedicated staff who implemented his or her orders at least occasionally.

The entire data on the percentage of reporting toxic leadership behaviors by nurse managers are detailed in Table 2.

### 3.3. Quality of Life of the Nurses

According to Table 3, over half of the nurses reported their overall quality of life as good (55.3%) and expressed satisfaction with their health (56.2%). In addition, 28.1% of the nurses rated their quality of life as very good, and 19.5% reported being very satisfied with their health. Only a small number of nurses reported their quality of life as poor (1.0%) or very poor (1.3%), and dissatisfaction with health was reported by 8.3% of the nurses, with 1.6% indicating they were very dissatisfied.

When examining the dimensions of quality of life, the highest scores were found in the “psychological health domain” (*M* = 77.96, SD = 15.85), followed by the “social relationships domain” (*M* = 75.55, SD = 20.10). In contrast, the lowest quality of life scores were reported in the “environment domain” (*M* = 67.81, SD = 19.09) and the “physical health domain” (*M* = 68.96, SD = 17.18).

### 3.4. Relationship Between the Nurses' Quality of Life and Nurse Managers' Toxic Leadership Behavior

Table 4 illustrates that there were weak negative correlations between the four dimensions of nurse managers' toxic leadership behaviors and three areas of well-being: “physical health” (*r* = −0.23 to −0.29, *p* < 0.001), “psychological health” (*r* = −0.22 to −0.26, *p* < 0.001), and “social relationships” (*r* = −0.16 to −0.23, *p* < 0.001). In addition, moderate negative correlations were found between the four dimensions of nurse managers' toxic leadership behaviors and the “environment domain” (*r* = −0.31 to −0.38, *p* < 0.001).

### 3.5. Influence of Nurse Managers' Toxic Leadership Behavior on the Nurses' Quality of Life

Multiple linear regression analyses were conducted to examine the influence of four dimensions of toxic leadership behaviors exhibited by nurse managers on various aspects of quality of life, while controlling for the demographic characteristics of the nurses. The four regression models were found to be significant, explaining 15.7% (*R*^2^ = 0.192, adjusted *R*^2^ = 0.157), 8.9% (*R*^2^ = 0.127, adjusted *R*^2^ = 0.089), 11.8% (*R*^2^ = 0.155, adjusted *R*^2^ = 0.118), and 16.0% (*R*^2^ = 0.195, adjusted *R*^2^ = 0.160) of the total variance in “physical health” (*F*_13_,_299_ = 5.47, *p* < 0.001), “psychological health” (*F*_13_,_299_ = 3.34, *p* < 0.001), “social relationships” (*F*_13_,_299_ = 4.21, *p* < 0.001), and the “environment domain” (*F*_13_,_299_ = 5.56, *p* < 0.001).

The regression models are presented in Tables 5, 6, 7, and 8. Based on the results, marital status, the hospital where the nurses work, and the toxic leadership behaviors of nurse managers significantly influenced the nurses' quality of life. Nurses who were married reported better outcomes in “physical health” (*β* = 5.79, *p*=0.014, 95% CI = 1.17 and 10.40), “psychological health” (*β* = 6.08, *p*=0.007, 95% CI = 1.66 and 10.51), and “social relationships” (*β* = 9.12, *p*=0.001, 95% CI = 3.59 and 14.64) compared to single nurses. Those who were separated, divorced, or widowed also experienced better “psychological health” (*β* = 6.33, *p*=0.038, 95% CI = 0.35 and 12.32) than their single counterparts.

Nurses working in Hospital 2 reported poorer “physical health” (*β* = −6.84, *p*=0.001, 95% CI = −10.75 and −2.93), “social relationships” (*β* = −6.92, *p*=0.004, 95% CI = −11.60 and −2.24), and “environment domain” (*β* = −5.35, *p*=0.016, 95% CI = −9.68 and −1.01) compared to nurses in Hospital 3. Conversely, nurses at Hospital 1 reported better “psychological health” (*β* = −5.83, *p*=0.039, 95% CI = −11.36 and −0.29) than those in Hospital 3.

Furthermore, a one-point increase in the mean score for “humiliating behavior” was associated with declines of 4.15 (*p*=0.019, 95% CI = −7.62 and −0.68), 4.67 (*p*=0.028, 95% CI = −8.83 and −0.51), and 5.47 (*p*=0.006, 95% CI = −9.32 and −1.62) in the mean scores for “physical health,” “social relationships,” and the “environmental domain,” respectively. These findings indicate that experiences of humiliating behavior from nurse managers are linked to poorer quality of life for nurses.

## 4. Discussion

This study aimed to explore the relationship between toxic leadership behaviors and nurses' quality of life, with a particular focus on how different leadership styles impact various domains of quality of life. The findings contribute to the growing body of literature that highlights the harmful effects of toxic leadership on employee health and well-being, especially in high-stress professions such as nursing.

### 4.1. Nurse Managers' Toxic Leadership Behaviors

The results from the ToxBH-NM scale indicate that, overall, nurses perceive their immediate supervisors' behavior positively, which is better than that reported by nurses from Ghana [17] and Iran [18]. This perception may reflect Kazakhstan's post-Soviet management traditions, characterized by strict hierarchies and authoritarian structures [19]. Despite ongoing reforms in nursing management, many nurses may still view questioning authority as disrespectful or risky, and negative comments about supervisors as insubordination [20]. Such cultural and systemic factors may normalize toxic behaviors.

Kazakhstan's continued development of nursing management structures [4] and reforms granting nurses greater responsibilities and autonomy [21] may also contribute to the generally favorable perception of nurse leaders. Improvements in job descriptions and leadership roles are largely viewed positively, reinforcing supportive attitudes toward nurse managers [4].

However, subscale analysis revealed instances of toxic behaviors. The highest scores were for “Humiliating behavior,” suggesting experiences of neglect or rude treatment. A similar finding was reported among nurses from the Philippines [22]. Elements of Soviet-era management, centralized, top-down authority and rigid protocols, can foster dictatorial behaviors, including public reprimands and lack of recognition [23, 24]. Challenging working conditions, such as high patient loads, inadequate staffing, and limited resources, may exacerbate such behaviors, creating an environment where nurses tolerate humiliation to avoid repercussions [25, 26].

The participants rated narcissistic behavior as the second-highest mean, which is the same as the findings reported in a study in Iran [18]. The nurses reported their nurse leaders as self-glorifying and prone to overestimating themselves. In various cultures, narcissism is sometimes encouraged, due in part to the emphasis on self-esteem, personal success, and status [27]. In Kazakhstan, the blend of traditional collectivism and emerging individualism may influence how nurses express their identities and view themselves in their profession [4]. These cultural values may exacerbate narcissistic tendencies as a method of coping with systemic issues. It has been observed that a nurse leader may demonstrate loyalty to the group to meet collectivist expectations while simultaneously seeking personal gains or admiration. This duplicity is often linked to narcissism, as it reflects an individual who wears a social mask to gain approval while satisfying their ego needs internally [28]. Therefore, nurse managers' narcissistic behavior is a trait that does not significantly contribute to toxic leadership.

The subscale with the lowest mean was “Self-promotional behavior,” similar to the studies in the Philippines (Labrague, 2021). This indicates that the nurses generally did not see their nurse leaders as excessively boastful or arrogant regarding their accomplishments. Consequently, self-promotion is not always viewed negatively by nurses. Instead, it can be seen as a natural part of asserting authority and demonstrating competence [29]. When nurse leaders highlight their achievements, it is often interpreted as a sign of a strong leader rather than as self-centeredness. However, Riisla et al. [30] noted that if a leader focuses solely on their personal achievements and goals, it can damage team cohesion. While confidence and a strong personality are essential for a nurse leader, they must also balance these traits with humility and regular acknowledgment of the team's accomplishments.

### 4.2. Relationship Between Nurse Managers' Toxic Leadership Behaviors and Nurses' Quality of Life

The results of this study indicate that nurses generally report a positive quality of life, suggesting a favorable perception of their overall well-being despite the well-documented challenges of healthcare work. This may be attributed to their strong professional identity, which enhances resilience, self-esteem, and motivation, particularly when reinforced by praise from patients, colleagues, and leaders [31]. Nurses' clinical training equips them with skills in emotional regulation, crisis management, and teamwork, enabling them to rationally navigate stressors and maintain a balanced perspective [32, 33]. This adaptability aligns with existing evidence that nurses derive optimism and resilience from their sense of purpose and the significance of their work [34].

Among the domains assessed, psychological health scored the highest, likely reflecting nurses' stress management skills and access to mental health support programs [35]. Nurses from Jordan [36] and Poland [37] likewise identified psychological health as the highest dimension of quality of life. Maintaining a positive psychoemotional state is essential for preventing burnout and ensuring sustainable professional performance [34]. Social relations also scored highly, reflecting the protective role of teamwork, social integration, and family support in buffering stress and reducing burnout risk [38].

Conversely, physical health and environment scored lowest, similar to the findings reported among nurses from the Philippines [14] and Saudi Arabia [39]. This finding underscores the impact of demanding work conditions such as long shifts, high physical demands, and limited control over schedules. These findings are consistent with research linking chronic stress, low physical activity, and poor working conditions to decreased physical well-being and life satisfaction [40, 41].

Despite the generally positive quality of life among nurses, the analysis revealed that marital status significantly influences their quality of life. Married nurses reported higher scores in physical health, psychological well-being, and social relationships compared to their single counterparts. This aligns with the findings of Mertika et al. [42], which indicates that having a spouse or partner can buffer against stress and emotional strain associated with professional activities. Family support, greater subjective stability, and emotional connections to personal life likely contribute to a more stable life and professional challenges [43]. Furthermore, marriage may lead to a more structured routine, reduced social isolation, and the ability to delegate some concerns, positively impacting physical and mental health.

In addition, the analysis of indicators from different institutions revealed significant variations in the quality of life among nurses working in various hospitals. Specifically, nurses at Hospital 2 reported lower levels of physical health, social relationships, and environmental satisfaction compared to those from Hospital 3. This discrepancy could be attributed to factors such as organizational climate, leadership styles, workload, and access to resources and support within the institution. This idea is consistent with findings from Hossny et al. [7], which emphasizes that toxic leadership and an unfavorable organizational climate significantly reduce satisfaction levels and affect employees' intentions to remain at their institutions. Moreover, Ruiz-Fernández et al. [9] highlight the relationship between working conditions and indicators of burnout and job satisfaction. Thus, the quality of the organizational environment and management style directly impacts various aspects of nurses' quality of life.

Another significant finding is the consistent and notable impact of humiliating behavior on three of the four quality of life domains: physical health, social relationships, and the environment. This suggests that toxic leadership negatively affects nurses' overall well-being. A probable explanation is that the humiliating actions of nurse leaders challenge professional dignity, personal competence, and relational trust, which are essential for functioning effectively in high-stress healthcare settings [44]. When these fundamental aspects are compromised, psychological stress can manifest as fatigue, insomnia, and headaches [45]. This, in turn, weakens interpersonal relationships within teams, decreases job satisfaction, and may lead to higher staff turnover [46]. Moreover, prolonged exposure to humiliating behavior can diminish the organizational support, fostering feelings of isolation and burnout and further deteriorating the organizational environment [47].

### 4.3. Limitations of the Study

This study has some limitations. First, the design of the study did not allow for testing cause-and-effect relationships. Therefore, we cannot conclude that nurses' experiences of toxic behavior from their managers directly impact their quality of life. Second, the sampling technique and the settings of the study limit the generalizability of the findings, so caution should be exercised when interpreting and applying these results in other contexts. Third, the study did not investigate the reasons behind the positive leadership behaviors of nurse managers. Future research should explore this issue using qualitative analysis to provide deeper insights into the discussion.

## 5. Conclusion

The study revealed that while nurses generally held positive views about the leadership behaviors of their managers, they also experienced toxic leadership behaviors. Some nurses reported instances where their managers raised their voices, repeatedly reminded them of their mistakes, belittled them, and punished the entire unit for the error of a single staff member. In addition, some nurses felt that their nurse manager believed he or she was always right and operated with a group of devoted staff who simply executed their orders. These humiliating behaviors from nurse managers were found to negatively impact the nurses' quality of life.

### 5.1. Implications to Nursing Management

The study provides compelling evidence on the detrimental impact of nurse managers' toxic leadership behaviors on nurses' quality of life. Such behaviors significantly compromise the well-being of nurses in clinical settings, leading to increased stress, burnout, and job dissatisfaction. Therefore, hospital and nursing leaders must develop and implement strategies that foster healthy and supportive leadership practices. Hospital policymakers should establish policies that explicitly address toxic leadership behaviors while promoting positive, empathetic, and respectful management approaches. The findings highlight the urgent need for leadership styles that prioritize empathy, respect, and support, as these can enhance nurses' well-being, reduce burnout and staff turnover, and ultimately improve patient care outcomes.

Investing in leadership development programs should remain a key priority, as these initiatives are essential for cultivating sustainable and effective human resources in healthcare. Such programs must specifically target the toxic leadership behaviors identified in the study and provide nurse managers with the skills, resources, and support needed to adopt positive leadership styles. It is also crucial to investigate the underlying factors that contribute to toxic leadership behaviors among nurse managers. Understanding these root causes can inform the design of interventions and support systems that help managers transition toward more constructive and empowering leadership practices.

## Figures and Tables

**Table 1:** Demographic characteristics of the respondents (*n* = 313).

Variables	Minimum	Maximum	Mean	SD
Age	19.00	64.00	33.08	11.62
Years of experience as a nurse	0.08	46.00	11.06	11.06
Years of experience as a nurse in the current hospital	0.08	42.00	6.01	6.08

	***n***	**%**		

Gender				
Male	12	3.8		
Female	301	96.2		
Marital status				
Single	143	45.7		
Married	124	39.6		
Separated/divorced/widow/er	46	14.7		
Education				
Certificate in nursing or midwifery	188	60.1		
Feldsher	35	11.2		
Baccalaureate in nursing	90	28.8		
Hospital				
Hospital 1	41	13.1		
Hospital 2	127	40.6		
Hospital 3	145	46.3		

**Table 2:** Results on the descriptive analyses on the toxic leadership behaviors of nurse managers scale (*n* = 313).

Item	Mean	SD	Not at all	Once in a while	Sometimes	Fairly often	Frequently
			*n* (%)	*n* (%)	*n* (%)	*n* (%)	*n* (%)
1. My nurse manager takes a stand against the staff without listening to them first	1.71	1.10	198 (63.3)	48 (15.3)	41 (13.1)	13 (4.2)	13 (4.3)
2. My nurse manager raises voice when his/her point is not favored or accepted by the staff	1.77	1.09	183 (58.1)	62 (19.8)	39 (12.5)	20 (6.4)	10 (3.2)
3. My nurse manager shows arrogance to his/her staff	1.50	0.95	226 (72.2)	44 (14.1)	24 (7.7)	12 (3.8)	7 (2.2)
4. My nurse manager causes staff to try to “read” his/her temper	1.49	0.95	234 (74.8)	28 (8.9)	34 (10.9)	11 (3.5)	6 (1.9)
5. My nurse manager blames the staff to save him/herself from shame	1.45	1.02	250 (79.9)	20 (6.4)	18 (5.8)	15 (4.8)	10 (3.2)
6. My nurse manager is rude and disrespectful to staff	1.41	0.90	224 (78.0)	32 (10.2)	22 (7.0)	8 (2.6)	7 (2.2)
7. My nurse manager allows his/her mood to command the climate of the unit or ward	1.62	1.07	212 (67.7)	45 (14.4)	31 (9.9)	13 (4.2)	12 (3.8)
8. My nurse manager does not value his/her staffs' contribution to the organization	1.63	1.12	217 (69.3)	38 (12.1)	27 (8.6)	18 (5.8)	13 (4.2)
9. My nurse manager belittles his/her staffs' work	1.70	1.16	208 (66.5)	37 (11.8)	39 (12.5)	12 (3.8)	17 (5.4)
10. My nurse manager initiates conflict among his/her staff	1.40	0.86	242 (77.3)	34 (10.9)	24 (7.7)	8 (2.6)	5 (1.6)
11. My nurse manager disregards ideas of his/her staff that are contrary to his/her own	1.47	0.97	235 (75.1)	38 (12.1)	20 (6.4)	11 (3.5)	9 (2.9)
12. My nurse manager easily gets annoyed when being questioned by her/his staff	1.51	0.94	221 (70.6)	49 (15.7)	27 (8.6)	8 (2.6)	8 (2.6)
13. My nurse manager ignores his/her staff as if they do not exist	1.40	0.89	246 (78.6)	33 (10.5)	17 (5.4)	11 (3.5)	6 (1.9)
14. My nurse manager punishes the entire unit for mistakes made by one staff member	1.65	1.08	206 (65.8)	45 (14.4)	37 (11.8)	14 (4.5)	11 (3.5)
15. My nurse manager does not trust anyone else to complete tasks effectively	1.45	0.93	238 (76.0)	32 (10.2)	28 (8.9)	7 (2.2)	8 (2.6)
16. My nurse manager believes that the future of the unit or ward only goes well with him/her	1.42	0.94	250 (79.9)	21 (6.7)	26 (8.3)	7 (2.2)	9 (2.9)
17. My nurse manager thinks he/she is always right	1.69	1.20	215 (68.7)	33 (10.5)	32 (10.2)	13 (4.2)	20 (6.4)
18. My nurse manager believes that he/she is an extraordinary person	1.47	1.00	240 (76.7)	30 (9.6)	23 (7.3)	9 (2.9)	11 (3.5)
19. My nurse manager cares only for his/her own ward/unit and not for others	1.47	0.97	235 (75.1)	39 (12.5)	21 (6.7)	7 (2.2)	11 (3.5)
20. My nurse manager believes that he/she deserves the position that he/she is in to the full extent	1.81	1.32	209 (66.8)	30 (9.6)	27 (8.6)	20 (6.4)	27 (8.6)
21. My nurse manager places his/her personal interest ahead of others	1.52	1.09	244 (78.0)	16 (5.1)	27 (8.6)	12 (3.8)	14 (4.5)
22. My nurse manager declines to share the accountability for the mistakes that the staff make	1.48	0.99	236 (75.4)	34 (10.9)	22 (7.0)	11 (3.5)	10 (3.2)
23. My nurse manager has a group of dedicated staff who implement his/her orders	1.73	1.24	214 (68.4)	31 (9.9)	27 (8.6)	22 (7.0)	19 (6.1)
24. My nurse manager lies to his/her staff to get his/her way	1.38	0.92	254 (81.2)	25 (8.0)	17 (5.4)	8 (2.6)	9 (2.9)
25. My nurse manager changes his/her behavior swiftly when his/her supervisor is present	1.55	1.07	228 (72.8)	37 (11.8)	20 (6.4)	16 (5.1)	12 (3.8)
26. My nurse manager employs deception to look good to his/her superiors	1.37	0.86	251 (80.2)	28 (8.9)	20 (6.4)	8 (2.6)	6 (1.9)
27. My nurse manager only treats favorably those staff who bear profit for him/her	1.60	1.10	220 (70.3)	40 (12.8)	28 (8.9)	9 (2.9)	16 (5.1)
28. My nurse manager repeatedly reminds staff of their previous mistakes	1.72	1.14	198 (63.3)	52 (16.6)	33 (10.5)	14 (4.5)	16 (5.1)
29. My nurse manager speaks negatively about his/her staff to other staff in the workplace	1.54	1.09	235 (75.1)	29 (9.3)	24 (7.7)	9 (2.9)	16 (5.1)
30. My nurse manager repeatedly reminds his/her staff that they are incompetent and inefficient at work	1.57	1.10	224 (71.6)	40 (12.8)	26 (8.3)	5 (1.6)	18 (5.8)

**Table 3:** Quality of life of the nurses (*n* = 313).

Variables	*n*	%
Perceived quality of life		
Very poor	4	1.3
Poor	3	1.0
Neither	45	14.4
Good	173	55.3
Very good	88	28.1
Satisfaction with health		
Very dissatisfied	5	1.6
Dissatisfied	26	8.3
Neither	45	14.4
Satisfied	176	56.2
Very satisfied	61	19.5

**Table 4:** Correlation between the toxic leadership behavior of nurse managers and nurses' quality of life (*n* = 313).

Variables	Intemperate behavior		Narcissistic behavior		Self-promoting behavior		Humiliating behavior	
	*r*	*p*	*r*	*p*	*r*	*p*	*r*	*p*
Physical health domain	−0.27	< 0.001^∗∗∗^	−0.23	< 0.001^∗∗∗^	−0.25	< 0.001^∗∗∗^	−0.29	< 0.001^∗∗∗^
Psychological health domain	−0.26	< 0.001^∗∗∗^	−0.24	< 0.001^∗∗∗^	−0.22	< 0.001^∗∗∗^	−0.25	< 0.001^∗∗∗^
Social relationships domain	−0.20	< 0.001^∗∗∗^	−0.16	< 0.001^∗∗∗^	−0.16	< 0.001^∗∗∗^	−0.23	< 0.001^∗∗∗^
Environment domain	−0.36	< 0.001^∗∗∗^	−0.32	< 0.001^∗∗∗^	−0.31	< 0.001^∗∗∗^	−0.38	< 0.001^∗∗∗^

^∗∗∗^Significant at 0.001 level.

**Table 5:** Result of the multiple linear regression on nurses' physical health (*n* = 313).

Predictor variables	*β*	SE-*b*	Beta	*t*	*p*	95% confidence interval	
Age	0.34	0.20	0.23	1.65	0.099	−0.06	0.73
Marital status (reference: single)							
Married	5.79	2.34	0.16	2.47	0.014^∗^	1.17	10.40
Separated/divorced/widow/er	3.25	3.17	0.07	1.03	0.306	−2.99	9.49
Education (reference certificate in nursing or midwifery)							
Feldsher	1.76	2.97	0.03	0.59	0.553	−4.08	7.61
Baccalaureate in nursing	−1.38	2.10	−0.04	−0.66	0.512	−5.50	2.75
Hospital (reference: Hospital 3)							
Hospital 1	−1.83	2.93	−0.04	−0.62	0.533	−7.60	3.94
Hospital 2	−6.84	1.99	−0.20	−3.44	0.001^∗∗^	−10.75	−2.93
Years of experience as a nurse	−0.25	0.22	−0.16	−1.14	0.253	−0.68	0.18
Years of experience as a nurse in the current hospital	−0.02	0.22	−0.01	−0.08	0.940	−0.45	0.42
Toxic leadership behavior of nurse managers							
Intemperate behavior	−3.22	2.82	−0.16	−1.14	0.256	−8.77	2.34
Narcissistic behavior	3.11	2.65	0.16	1.17	0.242	−2.11	8.33
Self-promoting behavior	−0.88	2.25	−0.05	−0.39	0.695	−5.32	3.55
Humiliating behavior	−4.15	1.76	−0.25	−2.35	0.019^∗^	−7.62	−0.68

*Note:* The dependent variable was the mean of the physical health dimension. *β*, the unstandardized coefficient; SE-*b*, the standard error; *R*^2^* = *0.192; adjusted *R*^2^* = *0.157.

^∗^Significant at 0.05.

^∗∗^Significant at 0.01.

**Table 6:** Result of the multiple linear regression on nurses' psychological health (*n* = 313).

Predictor variables	*β*	SE-*b*	Beta	*t*	*p*	95% confidence interval	
Age	−0.03	0.19	−0.02	−0.16	0.874	−0.41	0.35
Marital status (reference: single)							
Married	6.08	2.25	0.19	2.71	0.007^∗∗^	1.66	10.51
Separated/divorced/widow/er	6.33	3.04	0.14	2.08	0.038^∗^	0.35	12.32
Education (reference certificate in nursing or midwifery)							
Feldsher	−0.91	2.85	−0.02	−0.32	0.750	−6.51	4.70
Baccalaureate in nursing	−1.20	2.01	−0.03	−0.60	0.552	−5.16	2.76
Hospital (reference: Hospital 3)							
Hospital 1	−5.83	2.81	−0.12	−2.07	0.039^∗^	−11.36	−0.29
Hospital 2	−3.12	1.91	−0.10	−1.64	0.103	−6.87	0.63
Years of experience as a nurse	0.11	0.21	0.07	0.51	0.610	−0.30	0.52
Years of experience as a nurse in the current hospital	−0.20	0.21	−0.08	−0.92	0.359	−0.62	0.22
Toxic leadership behavior of nurse managers							
Intemperate behavior	−3.61	2.71	−0.19	−1.33	0.184	−8.94	1.72
Narcissistic behavior	−0.42	2.54	−0.02	−0.17	0.868	−5.43	4.58
Self-promoting behavior	1.17	2.16	0.07	0.54	0.588	−3.08	5.42
Humiliating behavior	−2.23	1.69	−0.14	−1.32	0.189	−5.56	1.10

*Note:* The dependent variable was the mean of the psychological health dimension. *β*, the unstandardized coefficient; SE-*b*, the standard error; *R*^2^* = *0.127; adjusted *R*^2^* = *0.089.

^∗^Significant at 0.05.

^∗∗^Significant at 0.01.

**Table 7:** Result of the multiple linear regression on nurses' social relationships (*n* = 313).

Predictor variables	*β*	SE-*b*	Beta	*t*	*p*	95% confidence interval	
Age	0.14	0.24	0.08	0.58	0.563	−0.34	0.62
Marital status (reference: single)							
Married	9.12	2.81	0.22	3.25	0.001^∗∗^	3.59	14.64
Separated/divorced/widow/er	−1.77	3.80	−0.03	−0.47	0.641	−9.24	5.70
Education (reference certificate in nursing or midwifery)							
Feldsher	−3.25	3.55	−0.05	−0.92	0.361	−10.25	3.74
Baccalaureate in nursing	−3.01	2.51	−0.07	−1.20	0.232	−7.95	1.93
Hospital (reference: Hospital 3)							
Hospital 1	−6.61	3.51	−0.11	−1.88	0.060	−13.52	0.29
Hospital 2	−6.92	2.38	−0.17	−2.91	0.004^∗∗^	−11.60	−2.24
Years of experience as a nurse	−0.14	0.26	−0.08	−0.54	0.589	−0.65	0.37
Years of experience as a nurse in the current hospital	−0.17	0.27	−0.05	−0.64	0.526	−0.69	0.35
Toxic leadership behavior of nurse managers							
Intemperate behavior	−3.77	3.38	−0.16	−1.11	0.266	−10.42	2.89
Narcissistic behavior	2.50	3.18	0.11	0.79	0.432	−3.75	8.75
Self-promoting behavior	2.00	2.70	0.09	0.74	0.460	−3.31	7.30
Humiliating behavior	−4.67	2.11	−0.24	−2.21	0.028^∗^	−8.83	−0.51

*Note:* The dependent variable was the mean of the social relationships dimension. *β*, the unstandardized coefficient; SE-*b*, the standard error; *R*^2^* = *0.155; adjusted *R*^2^* = *0.118.

^∗^Significant at 0.05.

^∗∗^Significant at 0.01.

**Table 8:** Result of the multiple linear regression on nurses' environment domain (*n* = 313).

Predictor variables	*β*	SE-*b*	Beta	*t*	*p*	95% confidence interval	
Age	0.09	0.22	0.05	0.38	0.705	−0.36	0.53
Marital status (reference: Single)							
Married	5.11	2.60	0.13	1.97	0.050	−0.01	10.23
Separated/divorced/widow/er	0.18	3.52	0.00	0.05	0.958	−6.74	7.11
Education (reference certificate in nursing or midwifery)							
Feldsher	−3.82	3.30	−0.06	−1.16	0.248	−10.30	2.67
Baccalaureate in nursing	−1.42	2.33	−0.03	−0.61	0.541	−6.00	3.16
Hospital (reference: Hospital 3)							
Hospital 1	−1.53	3.25	−0.03	−0.47	0.638	−7.94	4.87
Hospital 2	−5.35	2.20	−0.14	−2.42	0.016^∗^	−9.68	−1.01
Years of experience as a nurse	−0.09	0.24	−0.05	−0.39	0.698	−0.57	0.38
Years of experience as a nurse in the current hospital	−0.19	0.25	−0.06	−0.78	0.434	−0.68	0.29
Toxic leadership behavior of nurse managers							
Intemperate behavior	−3.69	3.13	−0.16	−1.18	0.239	−9.86	2.47
Narcissistic behavior	−0.18	2.94	−0.01	−0.06	0.951	−5.97	5.61
Self-promoting behavior	1.68	2.50	0.08	0.67	0.502	−3.24	6.60
Humiliating behavior	−5.47	1.96	−0.29	−2.80	0.006^∗∗^	−9.32	−1.62

*Note:* The dependent variable was the mean of the environment dimension. *β*, the unstandardized coefficient; SE*-b*, the standard error; *R*^2^* = *0.192; adjusted *R*^2^* = *0.157.

^∗^Significant at 0.05.

^∗∗^Significant at 0.01.

## Data Availability

The data that support the findings of this study are available from the corresponding author upon reasonable request.
